# Quality of sleep and risk for obstructive sleep apnoea in ambulant individuals with type 2 diabetes mellitus at a tertiary referral hospital in Kenya: a cross-sectional, comparative study

**DOI:** 10.1186/s12902-017-0158-6

**Published:** 2017-02-06

**Authors:** Sairabanu Mohammed Rashid Sokwalla, Mark David Joshi, Erastus Olonde Amayo, Kirtida Acharya, Jared Ongechi Mecha, Kenneth Kipyegon Mutai

**Affiliations:** 1 Department of Clinical Medicine and Therapeutics, University of Nairobi (UoN), Nairobi, Kenya; 2 Kenyatta National Hospital (KNH), Nairobi, Kenya; 3 Clinical Epidemiology Unit, University of Nairobi, Nairobi, Kenya

**Keywords:** Disturbed sleep, Prevalence, OSA- obstructive sleep apnoea, QOS- quality of sleep, T2DM- type 2 diabetes

## Abstract

**Background:**

Sleep disorders are common and associated with multiple metabolic and psychological derangements. Obstructive sleep apnoea (OSA) is among the most common sleep disorders and an inter-relationship between OSA, insulin resistance, obesity, type 2 diabetes (T2DM) and cardiovascular diseases has been established. Prevalence of sleep disorders in Kenyans, particularly in individuals with T2DM is unknown. We thus aimed to determine prevalence of poor quality of sleep (QOS) and high risk for OSA, among persons with T2DM and determine their associations with socio-demographic and anthropometric variables.

**Methods:**

Utilising a Cross- Sectional Descriptive design, QOS and risk for OSA were determined in a randomly selected sample of patients with T2DM (cases) and an age and sex matched comparison group. The validated Pittsburgh Sleep Quality Index (PSQI) and Berlin Questionnaire (BQ) were used to measure QOS and risk for OSA respectively. Associations between poor QOS, high risk for OSA, and socio-demographic and anthropometric variables in cases were evaluated.

**Results:**

From 245 randomly selected persons with T2DM attending outpatient clinics, aged over 18 years, 22 were excluded due to ineligibility thus 223 were included in the analysis; 53.8% were females, mean age was 56.8 (SD 12.2) years and mean BMI was 28.8 kg/m^2^ (SD 4.4). Among them, 119 (53%, CI 95% 46.5–60.2) had poor QOS and 99 (44% CI 95% 37.8–50.9) were at high risk for OSA. Among 112 individuals in comparison group, 33 (29.5%, CI 95% 20.9–38.3) had poor QOS and 9 (8%, CI 95% 3.3–13.4) had high risk for OSA. Cases had a significantly higher probability for poor QOS [OR 2.76 (95% CI 1.7–4.4))] and high risk for OSA [OR 9.1 (95% CI 4.4–19.0)].

Higher waist circumference was independently associated with a high risk for OSA in cases.

**Conclusions:**

We demonstrate a high burden of sleep disturbances in patients with T2DM. Our findings may have implications for clinicians to screen for sleep disorders when assessing patients with T2DM and warranting further attention by practitioners and researches in this field.

**Electronic supplementary material:**

The online version of this article (doi:10.1186/s12902-017-0158-6) contains supplementary material, which is available to authorized users.

## Background

Type 2 diabetes mellitus (T2DM), a recognised cardiovascular disease risk factor, is reaching epidemic proportions in developing countries. According to the International Diabetes Federation, diabetes mellitus affects around 285 million people globally, and the number is expected to increase to 438 million by 2030, with two-thirds of all cases occurring in low to middle-income countries [1]. Type 2 diabetes mellitus constitutes up to 90% of all cases of diabetes mellitus globally [1]. In Africa, prevalence rate for diabetes mellitus in 2013 was estimated at 4.8% and is projected to rise to 5.3% by 2035 [1]. In Kenya the estimated population prevalence ranges from 4.2% in a multiethnic study involving both rural and urban Kenyans to 5.3% in a recent study conducted in the largest urban slum in Kenya [2, 3].

Type 2 diabetes mellitus can predispose to disturbed sleep patterns and disturbed sleep in turn can predispose to insulin resistance and T2DM [4]. In comparative studies, patients with T2DM have been found to have a higher prevalence of sleep disturbances compared to matched non- diabetic individuals [5].

Overweight and/ or obesity in T2DM are a major risk factor for sleep disturbances, particularly obstructive sleep apnoea (OSA). Other factors contributing to disturbed sleep in diabetes include: nocturia, autonomic and peripheral neuropathy, hypoglycaemia and hyperglycaemia [6, 7].

Presence of sleep disorders in patients with T2DM causes worsening of glycemic control and increased risk for cardiovascular morbidity and mortality [8–10]. Several mechanisms have been postulated to explain increased cardiovascular risk including: increased sympathetic activity, increased oxidative stress, low grade inflammation and endothelial dysfunction [9, 10].

Various studies on the prevalence and CVD risk association, of sleep disorders have been undertaken; however data from the African continent is scant. To the best of our knowledge, there is no published data available on the burden and impact of sleep disturbances in patients with T2DM from the Sub Saharan African region.

Therefore this study set out to determine the prevalence of poor quality of sleep (QOS) and prevalence of high risk for OSA in persons with T2DM and in an age and sex matched non- diabetic comparison group. We also aimed to determine associations of sleep disturbance with socio-demographic, clinical and anthropometric measures in persons with T2DM.

## Methods

The study was a cross- sectional descriptive study with a comparative arm and was carried out at the Kenyatta National Hospital (KNH), a national, tertiary referral hospital located in Nairobi, the capital city of Kenya. The comparison arm was included to serve as a benchmark to determine sleep disturbances in a non diabetic population since no prior studies have been conducted in the field of sleep medicine in Kenya.

The outpatient department of KNH operates two diabetes clinics. A daily mini clinic, attends to 25 to 30 patients per day, with care being offered by registered Medical Assistants (Clinical Officers). A Friday main clinic attends to 55 to 60 patients, and care is offered by diabetologists and specialist physicians, along with internal medicine residents.

Study subjects were included if they were equal to or above 18 years old, were literate in English/ Kiswahili and provided informed consent. We excluded pregnant women, individuals with known psychiatric and/ or neurologic disease, fluid overload states, night shift jobs and those consuming cigarette and/ or alcohol in the preceding month since these conditions are associated with sleep disturbances [11, 12]. In addition to the above, study cases were individuals attending the diabetic outpatient clinics during the study period who had a file recorded diagnosis of type 2 diabetes (T2DM) based on American Diabetes Association criteria [13].

Comparison group were age and sex matched individuals, not diagnosed with diabetes, with no symptoms suggestive of diabetes and had a random capillary blood sugar (RCBS) reading in non- diabetic range (below 11.1 mmol/l). The comparison group was recruited from relatives of patients attending other outpatient clinics and hospital staff members.

Sample size for cases was computed using the Daniel formula for prevalence studies [14]. Imputing a 16% prevalence of high risk for obstructive sleep apnoea as reported in a Korean study [15], and a 5% precision, a minimal sample size of 207 was arrived at.

Comparison group was recruited in a 2:1 ratio of cases to comparison group. A 2:1 rather than 1:1 ratio was opted by the researchers on account of logistic difficulties encountered in recruitment in a pilot study. Further, since this was a pioneer study, the aim of the comparison group was not to serve as a control per se but to provide a benchmark for the general population.

A two stage screening strategy was utilised to select cases. File records of patients booked to attend diabetes clinics were consecutively screened for age and recorded diagnosis eligibility criteria. Cases were then recruited randomly using simple random sampling and comparison group were recruited consecutively.

The study procedure and relevance was explained to the participants and any queries clarified. Using aseptic technique, random capillary blood sugar (RCBS) was then obtained from the comparison group by a fingertip skin prick using a validated glucometer (ACON- Acon Laboratories, Inc - AconLabs.com). Comparison group individuals with RCBS above 11 mmol/l or between 7.8 mmol/l and 11 mmol/l with symptoms of diabetes mellitus were excluded from the study and referred for follow-up assessment. Those recruited were explained further study procedures.

Data collection was done by the first author assisted by a study trained registered medical assistant. Training was done by the first author with regards to standard procedures for completion of the study proforma and measurement of anthropometric measures. Participants’ socio-demographic data (age, gender, marital status, employment status, level of education) and a targeted medical history (duration of diabetes since diagnosis and medication use) were taken by the first author and research assistants through direct patient interview. Duration of diabetes was determined from the file or where not available, by directly interviewing the patients. Anthropometric measurements (BMI, waist circumference, neck circumference, hip circumference and waist/ hip ratio) were then obtained using standard procedures recommended internationally [16]. Study subjects were then introduced to and requested to complete two self administered sleep questionnaires in either English or Kiswahili as per their language preference. The questionnaires are: Pittsburgh Sleep Quality Index (PSQI) and Berlin Questionnaire (BQ) [17, 18].

Pittsburgh Sleep Quality Index (PSQI) is a ten item questionnaire assessing quality of sleep (QOS) and identifies individuals as poor or good quality sleepers by calculating the sum score (global score; range 0 to 21)) of seven domains of sleep. Global score of >5 is considered as poor QOS. The PSQI is a validated tool; when assessed using clinical and clinimetric properties, and validated against polysomnography, it demonstrated sensitivity of 89.6% and specificity of 86.5% for identifying poor QOS [19]. It has also been validated against the General Health Questionnaire and wrist actigraphy [20, 21].

The Berlin Questionnaire (BQ) for assessing risk for obstructive sleep apnoea (OSA) is a nine item questionnaire, with five items on snoring, three on daytime somnolence and one item on hypertension/ BMI. The snoring or daytime somnolence categories are positive if responses indicate persistent symptoms (greater than three to four times a week) on the questionnaire items. To score positive in the 3rd category a history of hypertension or a BMI greater than or equal to 25 kg/m2 is required. Positive scores in ≥2 categories identify individuals at high risk for OSA. The BQ has been validated against polysomnography, with a sensitivity of 86% and specificity of 77% for identifying individuals with OSA [22].

For purposes of non English speakers, a forward and backward translation of the validated questionnaires was undertaken by independent linguists and the forward translation was validated for content against the original English version [Additional files 1 and 2].

### Statistical analysis

Statistical Products and Service Solutions (SPSS) version 17.0 (vendor SPSS Inc., Chicago) was utilised. Data was summarized into means (standard deviation) and medians (inter quartile range) for continuous variables and proportions for categorical variables. We presented mean and standard deviation for normally distributed continuous data and compared between two groups using student’s *t*-test. The non- normally distributed data was presented using median and inter quartile range and compared between two groups using Mann Whitney *U* test.

The prevalence of poor quality of sleep (QOS) is presented in proportions with its corresponding 95% confidence intervals (CI). Risk for obstructive sleep apnoea (OSA) was categorized as high or low risk and presented as proportions (95% CI).

The association between categories of QOS and categorical variables were estimated using Odds Ratios and association with continuous variables was assessed using Student’s *t*-test.

Similarly, association between categories of risk for OSA and categorical variables was determined using odds ratios and association of high risk for OSA and continuous variables was assessed using Student’s *t*-test.

Multivariable logistic regression analysis was conducted to seek the independent correlates of poor QOS and high risk for OSA. All variables (socio demographic and anthropometric) with significant associations on univariable analysis were adjusted for in the logistic regression. All statistical tests were performed at 5% level of significance.

## Results

Between 8th October 2012 and 1st November 2012, out of 719 diabetic clinic attendees, 245 with a chart diagnosis of type 2 diabetes (T2DM) and aged over 18 years were randomly selected. On secondary screening, 17 were excluded: five were current smokers and/ or alcohol consumers, three were night shift workers, seven had language barriers; one refused consent and one had neurologic disease. Thus 228 participants were enrolled as study cases. Five cases had incomplete data, thus not included in the final analysis.

Comparison group individuals were recruited in the same time period as cases. From 126 age and sex matched non-diabetic individuals screened for comparison group eligibility from various sources (84 from relatives of patients attending other medical outpatient clinics and 42 from members of hospital staff), 13 were excluded (nine had random capillary blood sugar (RCBS) in diabetic range, two were smokers and or alcohol users, two were night shift workers and one refused consent). Thus 112 comparison group individuals (89% of those screened) were enrolled and included in final analysis.

### Demographics & disease characteristics

Mean age of the cases was 56.8 years (SD 12.2), 58.3% were females, 71.1% were urban residents and three quarter were married (75.8%); 79.8% were employed and majority (92.4%) had attained formal education (primary school and beyond), about two thirds (59.2%) of cases had completed secondary school and beyond (Table 1). The mean duration since diagnosis of T2DM was 10.4 years (SD 8.0). Mean BMI was 28.8 kg/m^2^ (SD 4.4, range 19.8–46.3). Almost two thirds of cases (62.8%) were on insulin therapy, either alone (13%) or in combination with oral anti-diabetic (OAD) drugs (49.8%) whereas 81 (36.3%) cases were on OAD alone (Table 1).

**Table 1:** Demographic, anthropometric and clinical characteristics of cases and comparison group

Variable	Cases	Comparison	*P* value
	n (%)	n (%)	
Age, mean (SD)	56.8 (12.2)	56.5 (11.7)	0.816
Gender			
Male	93 (41.7%)	46 (41.1%)	0.912
Female	130 (58.3%)	66 (58.9%)	
Current residence			
Urban	163 (73.1%)	100 (89.3%)	0.001
Rural	60 (26.9%)	12 (10.7%)	
Employment status			
Self employed	178 (79.8%)	103 (92.0%)	0.004
Dependant	45 (20.2%)	9 (8.0%)	
Level of education			
None	17 (7.6%)	16 (14.3%)	0.001
Primary	74 (33.2%)	38 (33.9%)	
Secondary	101 (45.3%)	29 (25.9%)	
Tertiary	31 (13.9%)	29 (25.9%)	
Marital status			
Single	18 (8.1%)	8 (7.1%)	0.626
Married	169 (75.8%)	92 (82.1%)	
Divorced	7 (3.1%)	2 (1.8%)	
Widowed	29 (13.0%)	10 (8.9%)	
Anthropometric measures, mean (SD)			
BMI (kg/m2)	28.8 (4.4)	25.8 (6)	0.001
Neck circumference (cm)	36.5 (3.2)	33.3 (2.9)	0.001
Waist circumference (cm)	100.3 (10)	90 (12.7)	0.001
Hip circumference (cm)	107.1 (9.1)	102.5 (12.3)	0.001
Waist/hip ratio	0.94 (0.07)	0.88 (0.07)	0.001
Duration of T2DM in years, median (IQR)	10 (4–15)	N/A	N/A
Medications			
Insulin	29 (13.0%)	N/A	N/A
Insulin and OAD	111 (49.8%)		
OAD alone	81 (36.3%)		
None	2 (0.9%)		

*BMI* body mass index, *T2DM* type 2 diabetes, *OAD* oral anti-diabetic drugs

The age and sex matched comparison group had a similar distribution as cases with regard to marital status, but significantly differed in residence, employment status and level of education. They also had significantly lower BMI, neck, waist and hip circumferences and waist/ hip ratio compared to cases (*p* = 0.001) (Table 1).

### Quality of sleep and risk for obstructive sleep apnoea

Poor Quality of Sleep (QOS) was detected in 53.4% (95% CI 46.5–60.2) of cases with a median global QOS score of 6.0 [Inter quartile range (IQR) 3–10]; and 44.4% (95% CI 37.8–50.9) of cases had high risk for obstructive sleep apnoea (OSA).

In the comparison group, 29.5% (95% CI 20.9–38.3) had poor QOS with a median global QOS score of 4.0 (IQR 2–6) and 8% (95% CI 3.3–13.4) had high risk for OSA.

Cases had significantly higher probability for poor QOS [OR 2.7 (95% CI 1.7–4.4)] and high risk for OSA [OR 9.1 (95% CI 4.4–19.0)] which remained significant after adjusting for obesity, residence, employment and education level [QOS adjusted OR 2.1 (95% CI 1.1–3.8); OSA adjusted OR 6.5 (2.9–14.6)] (Table 2).

**Table 2:** Odds for poor QOS & high risk for OSA in cases & comparison group

Variable	Cases	Comparison group	Crude		Adjusted for anthropometric/Socio- demographic measures^a^	
	n (%)	n (%)	OR (95% CI)	*P* value	OR (95% CI)	*P* value
Quality of sleep						
Poor	119 (53.4)	33 (29.5)	2.7 (1.7–4.4)	0.001	2.1 (1.1–3.8)	0.018
Good	104 (46.6)	79 (70.5)	1.0		-	
Risk for OSA						
High risk	99 (44.4)	9 (8.0)	9.1 (4.4–19.0)	0.001	6.5 (2.9–14.6)	<0.001
Low risk	124 (55.6)	103 (92.0)	1.0		-	

^a^Adjusted for residence, employment, education level and neck circumference

On univariable analysis to determine association between QOS and socio-demographic, anthropometric and clinical measures in cases, duration of diabetes since diagnosis and the risk of OSA were significantly associated with poor QOS (Table 3). Good quality sleepers (global QOS ≤5) had a longer median duration of T2DM than poor quality sleepers [10.0 years (range 5.0–15.0) vs 8.0 years (range 2.0–16.0), *p* = 0.033]. Similarly, 70.2% of the good quality sleepers had low risk of OSA compared to 42.9% of poor quality sleepers (p < 0.001). Adjusting for the two factors in multiple logistic regression (Table 4), duration of T2DM [OR 0.96 (95% CI 0.93–0.99), *p* = 0.021] and high risk for OSA [OR 3.4 (95% CI 1.9–5.9), *p* < 0.001] were independently associated with poor QOS in cases.

**Table 3:** Factors associated with poor QOS in cases

Variable	Quality of sleep (PSQI)		P value
	Good (*n* = 104)	Poor (*n* = 119)	
Age (mean (SD))	56.8 (12.2)	56.8 (12.2)	0.992
Gender			
Male	44 (42.3%)	49 (41.2%)	0.864
Female	60 (57.7%)	70 (58.8%)	
Marital status			
Single	7 (6.7%)	11 (9.2%)	0.607
Married	82 (78.8%)	87 (73.1%)	
Divorced	4 (3.8%)	3 (2.5%)	
Widowed	11 (10.6%)	18 (15.1%)	
Employment status			
Self employed	84 (80.8%)	94 (79.0%)	0.741
Dependant	20 (19.2%)	25 (21.0%)	
Medications			
Insulin	14 (13.5%)	15 (12.6%)	0.521
Insulin & OAD	53 (51.0%)	58 (48.7%)	
OAD	35 (33.7%)	46 (38.7%)	
None	2 (1.9%)	0 (0.0%)	
Duration of T2DM (years)	10.0 (5.0–15.0)	8.0 (2.0–16.0)	0.033
BMI (kg/m2) [mean (SD)]	28.7 (4.4)	28.8 (4.3)	0.969
Neck circumference	36.6 (3.1)	36.3 (3.3)	0.626
Waist circumference	100.0 (9.4)	100.5 (10.5)	0.523
Waist-hip ratio	0.93 (0.07)	0.94 (0.07)	0.308
OSA			
High risk	31 (29.8)	68 (57.1)	<0.001
Low risk	73 (70.2)	51 (42.9)	

*BMI* body mass index, *T2DM* type 2 diabetes, *OAD* oral anti-diabetic drugs

**Table 4:** Predictors of poor QOS in cases

Variable	OR (95% CI)	*P* value
Mean duration of T2DM (years)	0.96 (0.93–0.99)	0.021
High risk of OSA	3.4 (1.9–5.9)	<0.001

*T2DM* type 2 diabetes, *OAD* oral anti-diabetic drugs, *OSA* obstructive sleep

Compared to those at low risk for OSA, cases at high risk for OSA were more likely to be older (59 years vs 55.6 years; *p* = 0.017), unemployed (26.3% vs 15.3%; *p* = 0.001) and have higher mean waist circumference (102.2 [SD 10.7] cm vs 98.7 [SD 9.1] cm; *p* = 0.008) and hip circumference (108.7 [SD 10.0] cm vs 105.9 [SD 8.1] cm; *p* = 0.026). Cases at high risk for OSA were also more likely to have poor quality of sleep (68.7% vs 41.1%; *p <* 0.001) (Table 5). On multivariable analysis, higher waist circumference [OR1.04 (95% CI 1.01–1.07), *p* = 0.014] and poor QOS [OR 3.2 (95% CI 1.8–5.7), *p <* 0.001] were independently associated with a high risk for OSA (Table 6).

**Table 5:** Factors associated with high risk for OSA in cases

Variable	Berlin score		OR (95% CI)	P value
	High risk (*n* = 99)	Low risk (*n* = 124)		
Age	59.0 (12.2)	55.6 (11.8)	1.03 (1.01–1.05)	0.017
Gender				
Male	41 (41.4%)	52 (41.9%)	1.0 (0.6–1.7)	0.937
Female	58 (58.6%)	72 (58.1%)	1.0	
Marital status				
Single	10 (10.1%)	8 (6.5%)	1.0	
Married	73 (73.7%)	96 (77.4%)	0.6 (0.2–1.6)	0.319
Divorced	3 (3.0%)	4 (3.2%)	0.6 (0.1–3.5)	0.570
Widowed	13 (13.1%)	16 (12.9%)	0.7 (0.2–2.1)	0.475
Employment status				
Self employed	73 (73.7%)	105 (84.7%)	1.0	0.043
Dependant	26 (26.3%)	19 (15.3%)	2.0 (1.0–3.8)	
Duration of T2D (years)	10.0 (5.0–18.0)	10.0 (3.5–14.5)	1.01 (0.98–1.05)	0.405
Medications				
Insulin	17 (17.2%)	12 (9.7%)	2.1 (0.9–5.1)	0.084
Insulin and other	50 (50.5%)	64 (51.6%)	1.2 (0.7–2.1)	0.571
Others	31 (31.3%)	47 (37.9%)	1.0	
None	1 (1.0%)	1 (0.8%)	1.5 (0.1–25.1)	0.772
Neck circumference (cm)	36.7 (3.5)	36.2 (2.9)		0.270
Waist circumference (cm)	102.2 (10.7)	98.7 (9.1)		0.008
Hip circumference (cm)	108.7 (10.0)	105.9 (8.1)		0.026
Waist/hip ratio	0.94 (0.07)	0.93 (0.07)		0.380
Quality of PSQI score				
Good	31 (31.3%)	73 (58.9%)	1.0	<0.001
Poor	68 (68.7%)	51 (41.1%)	3.1 (1.8–5.5)	

*OAD* oral antidiabetic agents, *T2D* type 2 diabetes), *BMI* body mass index

**Table 6:** Predictors of high risk for OSA in cases (Logistic regression model)

Variable	OR (95% CI)	*P* value
Quality of PSQI score		
Good	1.0	<0.001
Poor	3.2 (1.8–5.7)	
Waist circumference	1.04 (1.01–1.07)	0.014
Employment status		
Self-employed	1.0	0.071
Dependent	1.9 (0.9–3.9)	

Age and hip circumference were significantly correlated to waist circumference hence excluded from the model

## Discussion

In this pioneer study in the field of sleep medicine in Kenya and utilising validated instruments, we demonstrate a high prevalence of sleep disorders in ambulatory persons with type 2 diabetes (T2DM). The obesity adjusted odds for poor QOS and high risk for OSA in study cases was two to three fold that of non-diabetic subjects. In addition, we demonstrate that high risk for OSA is independently associated with central obesity and poor sleep quality. Thus this study establishes a useful platform for conducting further research in sleep disturbances among persons with diabetes in Kenya.

To the best of our knowledge, no similar studies have been reported in African patients with T2DM. Utilising the PSQI and BQ, a Korean study, in patients with T2DM, report a 38% prevalence of poor QOS and 15.8% prevalence of high risk for OSA [15]. The lower prevalence rates in the Korean study compared to our study could be explained by study subjects’ low mean BMI (24.8Kg/ m^2^), exclusion of those on insulin for diabetes management and exclusion of patients with severe painful neuropathy. In our study the mean BMI was 28.8 Kg/m^2^, two third of our patients were on insulin therapy, and we did not exclude painful neuropathy, suggesting more complicated or poorly controlled diabetes subjects. However, from different parts of the world, prevalence rates for poor QOS in patients with T2DM, using the PSQI range from 38% in Korea to 69% in a South Asian Indian study [15, 23, 24]. Using subjects from the Sleep Heart Health study and utilising polysomnography, a high prevalence of OSA was found among patients with T2DM, where up to 58% had at least mild OSA (apnoea hypopnoea index (AHI) ≥5) [25].

Our finding of the association of obesity with OSA is consistent with previous reports. Obesity, older age and male sex have been shown to be independently associated with high risk for OSA in several reports [6, 25, 26]. Obesity increases the risk for OSA by mechanical obstruction, thus increasing upper airway resistance and increased tendency to develop apnoeic and hypopnoeic events during sleep [27]. In addition, fat deposits around the thoracic cage (truncal obesity) reduce chest compliance and functional residual capacity, thus may increase oxygen demand [28]. Further, genetic polymorphisms may influence both OSA and obesity, and may be interrelated in the development of both these conditions [29]. To assess the link between obesity and OSA, studies have shown that treating obesity by weight reduction improves OSA symptoms; a moderate weight reduction was shown to prevent the progression of mild OSA, and even cure it [30].

Although male sex is a recognized risk factor for OSA, explained by the increased mass in the torso and neck [31], we were unable to demonstrate such an association among our diabetic study subjects. Similar to our findings, a Turkish study utilising overnight polysomnograhy to assess gender differences in patients with OSA failed to find significant difference between sexes [32].

Older age was significantly associated with a high risk for OSA in our study, though it did not have an independent association. The prevalence of OSA increases with age; 2- to 3-fold higher prevalence in older persons (≥65 years) compared to middle aged persons (30–64 years) [33]. Old age is often accompanied by muscular and neurological loss of muscle tone of the upper airway with enhanced collapsibility, thus increasing risk for OSA [34, 35].

Our finding of poor QOS being inversely related to duration of diabetes diagnosis is a counter intuitive finding since we expected patients with longer duration of diabetes to have poorer quality of sleep due to increased complications. Most studies have reported a positive association between disease duration and poor QOS. A study on patients with T2DM in the USA, using the PSQI, reported that the poorest QOS was among patients who had been diagnosed more than 10 years earlier. This was attributed to more frequent diabetic complications after the first 10 years of disease, thus greater probability of having poor QOS [23]. A South East Indian study found a significant positive correlation between duration of T2DM and QOS, independent of other variables [24].

We are not aware of a plausible biological mechanism to explain our findings. A possible explanation could be that patients with longer duration of diabetes have better management of their conditions, resulting in better sleep and fewer symptoms. In addition, these could be spurious findings confounded by a measurement bias in establishing duration of disease.

### Strengths & limitations

Our study demonstrates certain strengths. To begin with this is the first reported study in the field of sleep medicine not only in Kenya but also from the East African region. Furthermore, we included a comparative arm to provide a reference baseline, undertook random sampling to select participants and used validated instruments.

Our limitations include: firstly, our results are from a tertiary referral hospital and are not generalisable to the entire T2DM Kenyan population. In addition, we used self reported questionnaires which depend heavily on factors such as the respondents’ mood, perception and level of education. Further, this was a cross- sectional study thus it is not possible to establish temporality between sleep disturbance and risk factors.

## Conclusion

We demonstrate a high burden of sleep disturbances in a high risk, previously unstudied, disease category of ambulatory subjects with T2DM. This calls for heightened awareness of clinicians to detect sleep disturbances in these patients. Furthermore, our findings warrant further research in the field of sleep disorders in diabetes including determining associations with quality of life, glycemic control and cardiovascular disease among others.

## Additional files

Additional file 1: Pittsburgh Sleep Quality Index: Kiswahili Translation. (DOC 52 kb)
Additional file 2: Berlin Questionnaire- Kiswahili Translation. (DOC 29 kb)

## Abbreviations

AHI: Apnoea hypopnoea index
BMI: Body mass index
BQ: Berlin Questionnaire
HC: Hip circumference
KNH: Kenyatta National Hospital
NC: Neck circumference
OSA: Obstructive sleep apnoea
PSQI: Pittsburgh Sleep Quality Index
QOS: Quality of sleep
T2DM: Type two diabetes mellitus
UK: United Kingdom
UoN: University of Nairobi
USA: United States of America
WC: Waist circumference
WHR: Waist hip ratio

## References

[1] International Diabetes Federation (2013). IDF Diabetes Atlas. Epidemiology and Morbidity. International Diabetes Federation. IDF Diabetes Atlas.

[2] Christensen DL, Friis H, Mwaniki DL, Kilonzo B, Tetens I, Boit MK, Omondi B, Kaduka L, Borch-Johnsen K (2009). Prevalence of glucose intolerance and associated risk factors in rural and urban populations of different ethnic groups in Kenya. Diabetes Res Clin Pract.

[3] Ayah R, Joshi MD, Wanjiru R, Njau EK, Otieno CF, Njeru EK, Mutai KK (2013). A population-based survey of prevalence of diabetes and correlates in an urban slum community in Nairobi, Kenya. BMC Public Health.

[4] Punjabi NM, Sorkin JD, Katzel LI, Goldberg AP, Schwartz AR, Smith P (2002). Sleep-disordered breathing and insulin resistance in middle-aged and overweight men. Am J Respir Crit Care Med.

[5] West SD, Nicoll DJ, Stradling JR (2006). Prevalence of obstructive sleep apnoea in men with type 2 diabetes. Thorax.

[6] Lamond N, Tiggemann M, Dawson D (2000). Factors predicting sleep disruption in type 2 diabetes mellitus. Sleep.

[7] Tantucci C, Scionti L, Bottini P, Dottorini ML, Puxeddu E, Casucci G, Sorbini CA (1997). Influence of autonomic neuropathy of different severities on the hypercapnic drive to breathing in diabetic patients. Chest.

[8] Yi-Wen T, Nai-Hsuan K, Tao HT, Chao YJ, Lin CJ, Chang KC, Chang SS, Chen JY (2012). Impact of subjective sleep quality on glycaemic control in type 2 diabetes. Fam Pract.

[9] Yaggi HK, Concato J, Kernan WN, Lichtman JH, Brass LM, Mohsenin V (2005). Obstructive sleep apnea as a risk factor for stroke and death. N Engl J Med.

[10] Tkacova R, Dorkova Z, Molcanyiova A, Radikova Z, Klimes I, Tkac I (2008). Cardiovascularrisk and insulinresistance in patients with obstructive sleepapnea. Med Sci Monit.

[11] Zhang L, Samet J, Caffo B, Punjabi NM (2006). Cigarette smoking and nocturnal sleep architecture. Am J Epidemiol.

[12] Stein MD, Friedmann PD (2005). Disturbed sleep and its relationship to alcohol use. Subst Abus.

[13] American Diabetes Association (2012). Standards of medical care in diabetes—2012. Diabetes Care.

[14] Daniel WW (1999). Biostatistics: a foundation for analysis in the health sciences.

[15] Unjin S, Hyejin L, Jee-Young O, Yeon-Ah S (2011). Sleep disorder and cardiovascular risk factors among patients with type 2 diabetes mellitus. Korean J Intern Med.

[16] World Health Organization (1989). Measuring obesity: classification and distribution of anthropometric data.

[17] Pittsburgh Sleep Quality Index. [https://www.gonzaga.edu/student-life/Health-Center/psqi_sleep_questionnaire_1_pg.pdf]. Accessed 3 Feb 2017.

[18] Berlin Questionnaire. [https://www.sleepapnea.org/assets/files/pdf/berlin-questionnaire.pdf]. Accessed 3 Feb 2017.

[19] Buysse DJ, Reynolds CF, Monk TH, Berman SR, Kupfer DJ (1989). The Pittsburgh sleep quality index: a new instrument for psychiatric practice and research. Psychiatry Res.

[20] Sherry AB, Adam PS, Anita S, Eric JK, Li-Yung L, Kristine E, Susan R, Sonia AI, Katie LS (2012). Validation of the Pittsburgh sleep quality index and the Epworth sleepiness scale in older black and white women. Sleep Med.

[21] Aloba OO, Adewuya AO, Ola BA, Mapayi BM (2007). Validity of the Pittsburgh Sleep Quality Index (PSQI) among Nigerian university students. Sleep Med.

[22] Netzer NC, Stoohs RA (1999). Using the Berlin Questionnaire to identify patients at risk for the sleep apnea syndrome. Ann Inter Med.

[23] Cunha MC, Zanetti ML, Hass VJ (2008). Sleep quality in type 2 diabetics. Rev Lat Am Enfermagem.

[24] Rajendran A, Parthsarathy S, Tamilselvan B, Seshadri KG, Shuaib M (2012). Prevalence and correlates of disordered sleep in Southeast Asian Indians with type 2 diabetes. Diabetes Metab J.

[25] Resnick HE, Redline S, Shahar E, Gilpin A, Newman A, Walter R, Ewy GA, Howard BV, Punjabi NM (2003). Sleep heart health study. Diabetes and sleep disturbances: findings from the sleep heart health study. Diabetes Care.

[26] Vgontzas AN, Papanicolaou DA, Bixler EO (2000). Sleep apnoea and daytime sleepiness and fatigue; relation to visceral obesity, insulin resistance and hypercytokinemia. J Clin Endocrinol Metab.

[27] Davies RJ, Ali NJ, Stradling JR (1992). Neck circumference and other clinical features in the diagnosis of obstructive sleep apnoea. Thorax.

[28] Naimark A, Cherniack RM (1960). Compliance of the respiratory system and its components in health and obesity. J Appl Physiol.

[29] Patel SR, Larkin EK, Redline S (2008). Shared genetic basis for obstructive sleep apnea and adiposity measures. Int J Obes.

[30] Tuomilehto H, Seppä J, Uusitupa M, Peltonen M, Martikainen T, Sahlman J, Kokkarinen K, Randell J, Pukkila M, Vanninen E, Tuomilehto J, Gylling H (2014). The impact of weight reduction in the prevention of the progression of obstructive sleep apnea: an explanatory analysis of a 5-year observational follow-up trial. Sleep Med.

[31] Jordan AS, Mc Evoy RD (2003). Gender differences in sleep apnea: epidemiology, clinical presentation and pathogenic mechanisms. Sleep Med Rev.

[32] Bozkurt MK, Oy A, Aydin D, Bilen SH, Ertürk IO, Saydam L, Ozgen F (2008). Gender differences in polysomnographic findings in Turkish patients with obstructive sleep apnoea syndrome. Eur Arch Otorhinolaryngol.

[33] Young T, Skatrud J, Peppard PE (2004). Risk factors for obstructive sleep apnea in adults. JAMA.

[34] Crow HC, Ship JA (1996). Tongue strength and endurance in different aged individuals. J Gerontol A Biol Sci Med Sci.

[35] Aviv JE, Martin JH, Jones ME (1994). Age-related changes in pharyngeal and supraglottic sensation. Ann Otol Rhinol Laryngol.

