# Supplementary material for: Quality of sleep and risk for obstructive sleep apnoea in ambulant individuals with type 2 diabetes mellitus at a tertiary referral hospital in Kenya: a cross-sectional, comparative study
Source: BMC Endocr Disord. 2017 Feb 6;17:7. doi: 10.1186/s12902-017-0158-6 (PMC5294825; doi:10.1186/s12902-017-0158-6)
Supplement: Additional file 1: — Pittsburgh Sleep Quality Index: Kiswahili Translation. (DOC 52 kb) [file 12902_2017_158_MOESM1_ESM.doc]

**Additional file 1: Pittsburgh Sleep Quality Index: Kiswahili Translation**

Jina: __________________________________________ Tarehe: ________________

**Maelekezo**: Maswali yafuatayo yanahusiana na tabia yako ya kawaida ya usingizi katika mwezi uliopita tu. Majibu yako yanahitaji kuonyesha jibu **sahihi zaidi** kwa wingi wa mchana na usiku katika mwezi uliopita. Tafadhali jibu maswali yote.

1. Katika mwezi uliopita, huwa unaingia kitandani usiku saa ngapi kwa kawaida?_________

2. Katika mwezi uliopita, kwa kawaida huwa inakuchukua muda gani (kwa dakika) kupata usingizi kila usiku? ___________________________________

3. Katika mwezi uliopita, kwa kawaida huwa unaamka asubuhi saa ngapi? ______________

4. Katika mwezi uliopita, ni masaa mangapi halisi ulilala kila usiku? (Hii inaweza kuwa tofauti na idadi ya masaa uliokuwa kitandani). ___________________

| 5.Katika mwezi uliopita, ni mara ngapi umepata shida kulala kwa sababu: | Sikuwa na shida kwa mwezi uliopita | Si zaidi ya mara moja kwa wiki | Mara moja au mbili kwa wiki | Mara tatu au zaidi kwa wiki |
| --- | --- | --- | --- | --- |
| 1. Hukuweza kupata usingizi katika dakika 30 za kwanza |  |  |  |  |
| 1. Uliamka katikati ya usiku au asubuhi mapema |  |  |  |  |
| 1. Ulihitaji kuamka kutumia msalalani/ choo |  |  |  |  |
| 1. Hukuweza kupumua vizuri |  |  |  |  |
| 1. Ulikohoa au kukoroma kwa sauti kubwa. |  |  |  |  |
| 1. Ulihisi baridi sana |  |  |  |  |
| 1. Ulihisi joto sana. |  |  |  |  |
| 1. Ulipata ndoto mbaya. |  |  |  |  |
| 1. Ulihisi uchungu. |  |  |  |  |
| 1. Sababu zingine ( tafadhali eleza zaidi): |  |  |  |  |
| 6. Katika mwezi uliopita, ni mara ngapi umetumia dawa za kukusaidia kulala (ilioagizwa na daktari au bila kuagizwa)? |  |  |  |  |
| 7. Katika mwezi uliopita, ni mara ngapi ulipata shida kukaa macho wakati wa kuendesha gari au kukula chakula au kufanya shuguli zako? |  |  |  |  |
|  | Sikuwa na tatizo lolote | Tatizo kidogo tu | Tatizo kiasi | Tatizo kubwa sana |
| 8. Katika mwezi uliopita, ulikuwa na kiasi gani cha tatizo la hamu ya kutenda shughuli zako? |  |  |  |  |
|  | Mzuri sana | Mzuri kiasi | Mbaya kiasi | Mbaya sana |
| 9. Katika mwezi uliopita, utapimaje kiwango/ubora wa usingizi wako kwa jumla? |  |  |  |  |
|  | Hapana | Yuko lakini hulala katika chumba kingine | Yuko Chumbani mwangu lakini hulala katika kitanda kingine | Kitandani mwangu |
| 10. Je, unatumia kitanda kimoja au chumba kimoja na mtu mwingine? |  |  |  |  |
|  | Sikuwa na shida kwa mwezi uliopita | Si zaidi ya mara moja kwa wiki | Mara moja au mbili kwa wiki | Mara tatu au zaidi kwa wiki |
| Ikiwa una mwenzako kwa kitanda kimoja au kwa chumba kimoja, muulize ni mara ngapi katika mwezi ulliopita ulikuwa na: |  |  |  |  |
| 1. Mkoromo kwa sauti kubwa |  |  |  |  |
| 1. Misimamo mrefu (kuacha kupumua)katika pumzi katika usingizi/ |  |  |  |  |
| 1. Miguu kupapatika au kutetemeka katika usingizi. |  |  |  |  |
| 1. Nyakati za kuchanganyikiwa unapoamka katikati ya usingizi. |  |  |  |  |
| 1. Nyakati za kutotulia kwa sababu zingine wakati ulipokuwa umelala, tafadhali eleza: |  |  |  |  |
