# Supplementary material for: Quality of sleep and risk for obstructive sleep apnoea in ambulant individuals with type 2 diabetes mellitus at a tertiary referral hospital in Kenya: a cross-sectional, comparative study
Source: BMC Endocr Disord. 2017 Feb 6;17:7. doi: 10.1186/s12902-017-0158-6 (PMC5294825; doi:10.1186/s12902-017-0158-6)
Supplement: Additional file 2: — Berlin Questionnaire- Kiswahili Translation. (DOC 29 kb) [file 12902_2017_158_MOESM2_ESM.doc]

**Additional file 2: Berlin Questionnaire- Kiswahili Translation**

**DODOSO BERLIN: (BERLIN QUESTIONNAIRE- KISWAHILI)**

Urefu (m) ________ uzito (kg) ________ umri______ Mwanaume / Mwanamke
Tafadhali chagua jibu sahihi kwa kila swali.
**Sehemu 1**
**1. Je una koroma?**
 a. Ndio   b. La  c. sijui
**Kama una koroma:**
**2. Mkoromo wako ni wa sauti:**
a. Kiasi kidogo zaidi kuliko sauti ya kupumua

b. Kama ya kuzungumza
c. Zaidi kuliko kuzungumza

d. Kubwa sana – inaweza kusikika katika vyumba vilivyo karibu
**3. Ni mara ngapi wewe hukoroma**
 a. Karibu kila siku. b. Mara 3-4 kwa wiki
 c. Mara 1-2 kwa wiki. d. Mara 1-2 kwa mwezi
 e. Kamwe au karibu kamwe
**4. Je mkoromo wako ume wahi kuwasumbua watu wengine?**
 a. Ndio b. La c. Sijui
**5. Kuna mtu yeyote aliyetambua unaacha kupumua katikati ya usingizi?**
 a. Karibu kila siku. b. Mara 3-4 kwa wiki
 c. Mara 1-2 kwa wiki d. Mara 1-2 kwa mwezi
 e. Kamwe au karibu kamwe

**Jamii 2**
**6. Ni mara ngapi unahisi uchovu baada ya kulala?** a. Karibu kila siku. b. Mara 3-4 kwa wiki
 c. Mara 1-2 kwa wiki. d. Mara 1-2 kwa mwezi
 e. Kamwe au karibu kamwe
**7. Katika muda wako wa uchao, unahisi uchovu, udhoofu ama kutoridhika?** a. Karibu kila siku. b. Mara 3-4 kwa wiki
 c. Mara 1-2 kwa wiki. d. Mara 1-2 kwa mwezi
 e. Kamwe au karibu kamwe
**8. Je, umewahi kuhisi usingizi au kusinzia unapoendesha gari?**
 a. Ndio b. La
Kama ndio:
**9. Hii hutokea mara ngapi?**
 a. Karibu kila siku. b. Mara 3-4 kwa wiki
 c. Mara 1-2 kwa wiki. d. Mara 1-2 kwa mwezi
 e. Kamwe au karibu kamwe
**Jamii 3**
**10. Je, una shinikizo la damu? (blood pressure/ hypertension)**a. Ndio  b. La c. Sijui
